# Evaluation of corrective measures implemented for the preventive conservation of fresco paintings in Ariadne’s house (Pompeii, Italy)

**DOI:** 10.1186/1752-153X-7-87

**Published:** 2013-05-17

**Authors:** Paloma Merello, Fernando-Juan García-Diego, Manuel Zarzo

**Affiliations:** 1 Department of Applied Physics (UD Agriculture Engineering), Universitat Politècnica de València, Camino de Vera s/n, Valencia, 46022, Spain; 2 Valencian Institute for Conservation and Restoration of Cultural Heritage, C/ Genaro Lahuerta 25-3°, Valencia, 46010, Spain; 3 Center of Physical Technologies, Associated Unit ICMM-CSIC/UPV, Universitat Politècnica de València, Av de los Naranjos s/n, Valencia, 46022, Spain; 4 Department of Applied Statistics, Operations Research and Quality, Universitat Politècnica de València, Camino de Vera s/n, Valencia, 46022, Spain

**Keywords:** Microclimate monitoring, Pompeii, Archaeological preservation, Temperature and relative humidity sensors

## Abstract

**Background:**

A microclimate monitoring study was conducted in 2008 aimed at assessing the conservation risks affecting the valuable wall paintings decorating Ariadne’s House (Pompeii, Italy). It was found that thermohygrometric conditions were very unfavorable for the conservation of frescoes. As a result, it was decided to implement corrective measures, and the transparent polycarbonate sheets covering three rooms (one of them delimited by four walls and the others composed of three walls) were replaced by opaque roofs. In order to examine the effectiveness of this measure, the same monitoring system comprised by 26 thermohygrometric probes was installed again in summer 2010. Data recorded in 2008 and 2010 were compared.

**Results:**

Microclimate conditions were also monitored in a control room with the same roof in both years. The average temperature in this room was lower in 2010, and it was decided to consider a time frame of 18 summer days with the same mean temperature in both years. In the rooms with three walls, the statistical analysis revealed that the diurnal maximum temperature decreased about 3.5°C due to the roof change, and the minimum temperature increased 0.5°C. As a result, the daily thermohygrometric variations resulted less pronounced in 2010, with a reduction of approximately 4°C, which is favorable for the preservation of mural paintings. In the room with four walls, the daily fluctuations also decreased about 4°C. Based on the results, other alternative actions are discussed aimed at improving the conservation conditions of wall paintings.

**Conclusions:**

The roof change has reduced the most unfavorable thermohygrometric conditions affecting the mural paintings, but additional actions should be adopted for a long term preservation of Pompeian frescoes.

## Background

The long-term preservation of wall paintings in open-air sites or semi-confined environments is a challenge due to the difficulty in providing optimum ambient conditions. In such cases, the deterioration process of paintings is determined by many factors such as petrographical and chemical characteristics of the materials, presence of mineral salts and organic substances on the surfaces, air pollution, sunlight, heating, water content of the surface, etc. [1].

Weathering and disintegration of buildings, masonries and artifacts, as a result of salt efflorescence effects, have been widely studied. Rocks undergo deterioration processes due to temperature changes because most salts have high coefficients of volumetric expansion [2]. Moisture availability and insolation are also climatic variables affecting weathering [3]. Nonetheless, reported evidence indicates that the atmosphere has little corrosive effect on stone in the absence of water. Thus, it is important to monitor the presence of rainwater when assessing the damages caused by weathering in materials of different compositions [4-7]. Wide diurnal temperature fluctuations, sun intensity and sporadic heavy rain showers are the propelling factors for the chemical weathering [8].

Wall paintings are very sensitive to multiple factors such as (i) climatic conditions, especially temperature and humidity [9,10], (ii) presence of soluble salts, (iii) microbiological activity, and (iv) external factors like vandalism or tourism [10]. Indoor environments are more appropriate for the conservation of wall paintings because rainwater is rarely a problem and climatic conditions can be controlled. Many works have monitored thermohygrometric parameters inside museums for the preventive conservation of their collections [11-13] as well as in churches [14-17], but few studies have characterized ambient conditions in semi-confined [18] or open-air archaeological sites [10,19].

The house of Ariadne or *dei capitelli colorati* (of the colored capitals) is one of the most interesting places in ancient Pompeii (Italy). It is located at less than 100 m from the forum (Regio VII, *insula* 4) and presents a surface of 1,700 m^2^[20], being one of the largest *domus* of Pompeian architecture. A 3-D view of the place obtained from a photogrametric scan of the whole ruins [21] shows the remarkable quality of some wall paintings. Detailed pictures of all lodgings in Ariadne’s house are available [22].

**Figure 1:**
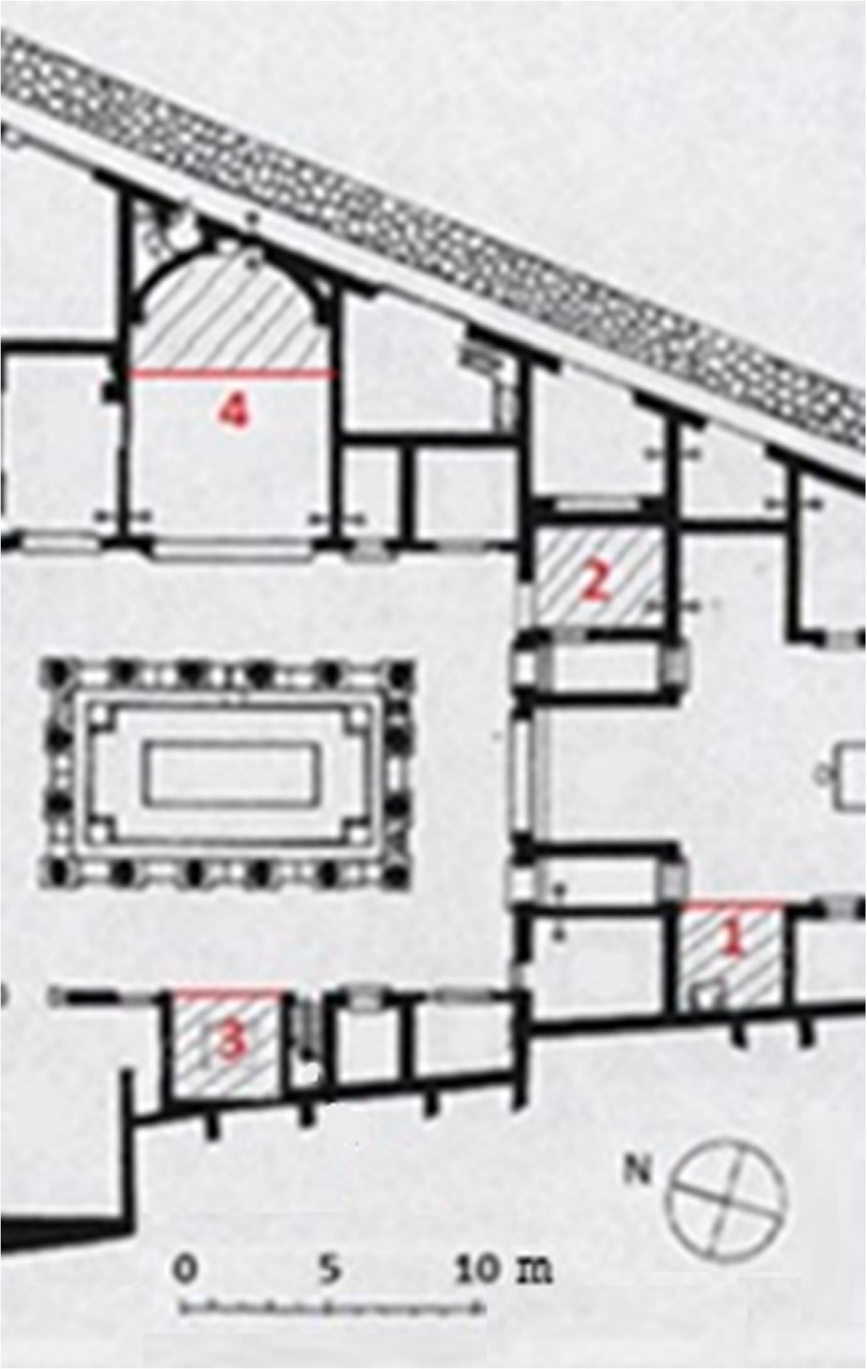
**Plan of Ariadne’s house displaying the four rooms under study.** Parallel sloping lines define the roofed area. A more detailed plan of the whole house with pictures of each room is available in [22].

Although most interior walls were originally ornamented with frescoes, the paintings have suffered severe damages since the excavation of Ariadne’s house in 1832–1835. At present, original frescoes are only conserved in three rooms that were sheltered with transparent polycarbonate sheets in the 1970s (coded as 1–3 in Figure 1), and in one additional lodging (room 4, *apsidal exedra*) that was covered in the 1950s with a roof of ceramic tiles (see [22], web link to room number 29). Room 3 (*exedra*, coded as lodging 18 in [22]) displays a mosaic of Hellenistic inspiration on the floor (84 × 77 cm) protected with a glass box. The mosaic is probably from the second century BC, and the rest of the floor is paved with tiles of a different style (first century AD). Room 1 (west side of *atrium*, lodging 6 in [22]) is delimited by three walls, as well as rooms 1 and 4. By contrast, room 2 (*oecus*, coded as 12 in [22]) is composed of four walls.

Mural paintings of Ariadne’s house have undergone deterioration processes in the last decades, and a research project was launched in 2008 to assess their conservation state by means of microclimate monitoring, thermography, study of materials, solar radiation, characterization of salt efflorescence, etc. [23]. With respect to the microclimate monitoring, a set of 26 thermohygrometric probes were installed in July 2008 inside the covered rooms (Table 1). Each probe was composed of one relative humidity (RH) data-logger and one temperature data-logger. It was found that the transparent roofs produced an unfavorable greenhouse effect causing excessive temperatures, particularly in summer [24]. As a result, the covering of rooms 1–3 was replaced in December 2009 by undulating opaque red roof sheets made of fiber cement with a thickness of about 6 mm, model ColorAGRI® rosso of Edilit SpA (Padova, Italy) [25].

**Table 1:** Position of thermohygrometric probes

**Code**	**Height**^**a**^	**Room**	**Pictures**^**b**^	**Code**	**Height**^**a**^	**Room**	**Pictures**^**b**^
#3	153	4	3^th^, 4^th^ at L29	#15	30	2	7^th^ at L12
#4	290	4		#16	0	2	12^th^ at L13
#5	0	3	1^st^ at L18	#17	338	2	4^th^, 5^th^, 8^th^ at L13
#6	0	3	1^st^ at L18	#18	300	2	7^th^ , 10^th^ at L12
#7	163	3	1^st^ , 6^th^ at L18	#19	310	2	7^th^ , 9^th^ at L12
#8	0	3	1^st^, 2^nd^, 3^rd^ at L18	#20	290	2	7^th^ at L12
#9	189	3	1^st^, 3^rd^, 6^th^ at L18	#21	175	1	2^nd^, 3^rd^, 17^th^ at L6
#10	340	3	1^st^ , 6^th^ at L18	#22	240	1	3^rd^, 11^th^, 12^th^ at L6
#11	0	3	1^st^ , 8^th^ at L18	#23	330	1	3^rd^, 15^th^ at L6
#12	210	3	6^th^ , 8^th^ at L18	#24	117	1	2^nd^, 3^rd^, 15^th^ at L6
#13	0	2	11^h^ at L13	#25	54	1	3^rd^, 10^th^, 15^th^ at L6
#14	0	2	2^nd^ at L13	#26	15	1	10^th^, 11^th^, 13^th^ at L6

^a^Distance to the ground level in cm. Probes #6, #8 and #11 were covered with a ceramic tile that can be seen in [22]. Probe #5 was located inside the glass box displayed in lodging 18 of [22].

^b^Pictures in [22] showing the exact position. For example, probe #7 appears in the 1^st^ and 6^th^ pictures displayed in [22] after following the link of lodging 18 (L18), which is coded as room 3 in Figure 1. Lodgings (L) numbered as 6, 12 and 29 in the room plan of [22] (coded here as L6, L12 and L29) correspond to rooms 1, 2 and 4, respectively. The link to lodging 13 (L13) in [22] also shows pictures of room 2.

Structural details about the initial roof in rooms 1 and 3 can be seen in [22], following the link to lodgings 6 (1^st^ and 3^rd^ pictures) and 18 (2^nd^ picture). The photographs were taken in March 2009 and show the roof slope as well as the metallic structure supporting the polycarbonate sheets. The water drainage gutter and downspout can also be observed. The uniform height of walls propitiated the roof installation directly fixed to the room upper perimeter (see p. 97 of [26]), leaving a negligible ventilation space through the roof borders (less than 5 cm between the shelter and wall top). Such reduced space is not a problem in this case because rooms 1 and 3 are composed of three walls, which allows an appropriate ventilation. In room 2, the initial roof was also directly resting on the top of the four walls delimiting this lodging as shown in [22], following the link to lodgings 12 (12^th^ picture) and 13 (5^th^ picture).

The roof change of room 1 performed at the end of 2009 is clearly illustrated by comparing the 3^rd^ picture of lodging 6 [22] with a photograph of this room taken in 2010 [23]. The latter shows that three supporting metal square tubes were installed perpendicular to the existing ones to fix conveniently the fiber cement sheets. It can also be noticed in this picture that the roof is not perfectly sealed to the wall borders, allowing certain ventilation. The distance between the wall top and the sheets is about 10 cm. Exhaustive details concerning the design of complex shelters mounted on open-air archaeological sites are described for the Villa Arianna at Castellammare di Stabia (see [26], pp. 307–312) and Punta d’Alaca in the Italian island of Vivara (see [26], pp. 319–323). A comprehensive description of the whole covering project is not addressed here because the shelters are relatively simple and the pictures in [22] provide clear information.

In order to assess the effectiveness of the roof change, the data-loggers used in the previous study [24] were installed in the same locations in summer 2010. The present work performs a comparative statistical analysis of data recorded in 2008 and 2010 (summer periods) aimed at evaluating the effect of roof change on the microclimate conditions surrounding the valuable fresco paintings. Results provide guidelines for additional corrective measures.

## Results and discussion

### Monitoring periods

As the summer season is the most hostile for the conservation of outdoor wall paintings given the high temperatures and daily variations, it was decided to conduct the study in this season. The monitoring period started on July 20^th^ 2010 and ended on September 15^th^ 2010, resulting a frame of 58 days.

Each temperature data-logger was paired with a RH data-logger by means of a PVC structure. This assembly will be referred to hereafter as thermohygrometric probe (coded as #1 to #26 in Table 1). All probes were placed inside the 4 rooms under study except #1, which was located on the top of an outside wall to serve as a control. Unfortunately, data-loggers of probes #1 and #2 were wrongly programmed and they were disregarded.

**Figure 2:**
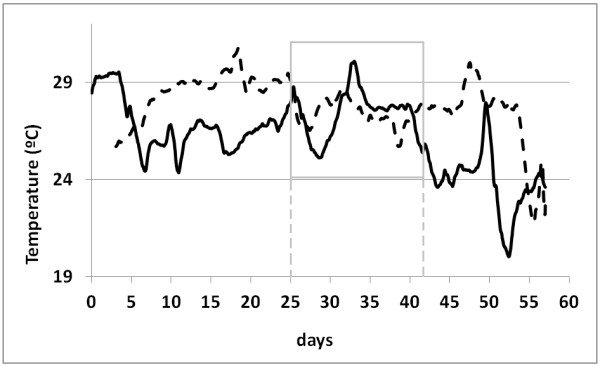
**Average temperature in summer 2008 and 2010 recorded by data-loggers #3 and #4 (room 4).** Day 0 corresponds to July 20^th^ 2008 (dashed trajectory) and July 20^th^ 2010 (continuous). A moving average with a window size of 48 data (i.e., one day) was applied to smooth both time series. The time frame from day 25 to 42 (highlighted in gray color) presents a similar average temperature in both years.

Data recorded in 2008 and 2010 can only be directly compared in the case of similar thermohygrometric conditions outside the rooms. Meteorological data from a weather station in Pompeii would be necessary to check this issue. Unfortunately, the closest stations are located in Naples and Capri, too far away. Given that room 4 was the only one that maintained the same roof, probes located there (#3 and #4) can be used as a reference to compare ambient conditions in both years. Figure 2 shows the average trajectories of temperature in room 4 for the monitored period of 58 days. In this period, the mean temperature was 27.8°C in 2008 and 26.2°C in 2010. The fact that summer 2008 was hotter may lead to a misinterpretation of data from probes in rooms 1–3. Thus, it is not possible to conclude if the observed differences of temperature in rooms 1–3 are caused by the roof change or are due to the different outside temperatures of each year, which implies that both effects are confounded.

**Figure 3:**
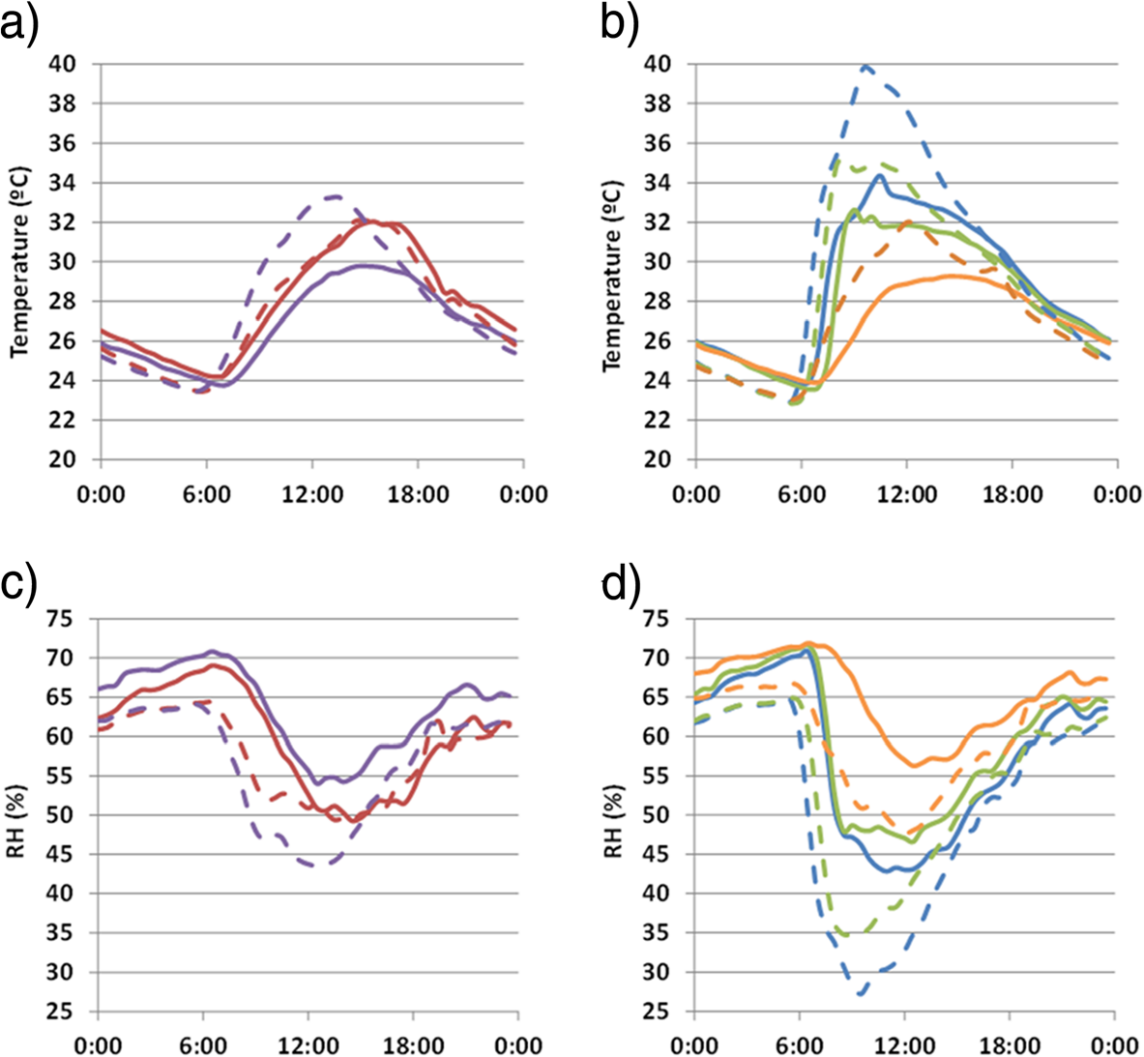
**Mean daily trajectories of temperature and RH in summer.** Figures (**a**, **b**) depict temperature trajectories and (**c**, **d**) RH trajectories. Monitoring period: 14–31 August 2008 (dashed trajectories); 14–31 August 2010 (continuous). Trajectories were averaged for all data recorded in each room except by floor probes. Color codes: green (probes in room 1: #21 - #26); violet (room 2: #15, #17 - #20); blue (room 3: #7, #9, #10, #12); red (room 4: #3, #4); orange (floor probes: #6, #8, #11, #13, #14, #16). The probe inside the mosaic glass box (#5) was not considered here.

Attempting to avoid this confusion of effects, it was decided to select a time frame of the monitoring period with a similar average temperature in both years inside room 4. The interval chosen was August 14^th^ to 31^st^ (18 days), as indicated in Figure 2. For probes in room 4, Figure 3a shows that the mean diurnal evolution of temperature in the 18-day period was nearly the same in 2008 and 2010. Moreover, the average RH was also similar in both years (Figure 3c). Thus, it was assumed that outside ambient conditions were similar in this time frame of both years. Data out of this interval were disregarded.

The time series recorded by each data-logger reflects the evolution of the measured parameter versus time, and it is commonly denoted as trajectory. By carefully inspecting all trajectories recorded in 2008, it was reported in the previous study [24] that certain probes underwent abnormal peaks of temperature at particular time frames, which was caused by solar radiation incident on the probes. Trajectories obtained in 2010 were also visually examined, and only probe #15 yielded a typical temperature peaks from about 5:00 PM to 8:00 PM caused by sunshine entering through the entryway of room 2. The abnormal data were removed.

### Mean daily trajectories

The average daily trajectories recorded by data-loggers in the selected 18-day period are displayed in Figure 3, which provides useful information to discuss the effect of roof change on thermohygrometric conditions. Minimum temperatures of each probe were similar in both years, but the maximum values in rooms 1–3 were obtained in 2008. Similarly, trajectories of RH are also more pronounced in 2008. The microclimate was less hot and less dry after the roof change, which is more favorable from a preservation standpoint.

There is a lack of consensus about the ideal or limit values of thermohygrometric parameters for an optimum maintenance of frescoes. The Italian standard DM 10/2001 [27] indicates reference values for the conservation of cultural heritage. It does not provide guidelines for outdoor paintings, but nonetheless the admissible values suggested by this standard for indoor mural paintings (RH: 45 – 60% and temperature: 6 – 25°C) can be taken as a reference for the present work.

Figures 3c and 3d show that RH values below 45% were recorded at midday in rooms 1–3 in 2008. By contrast, such dry conditions did not occur in 2010 except in room 3. Thus, the roof change has avoided the low RH registered in 2008 that could be regarded as harmful for the frescoes according to [27].

**Figure 4:**
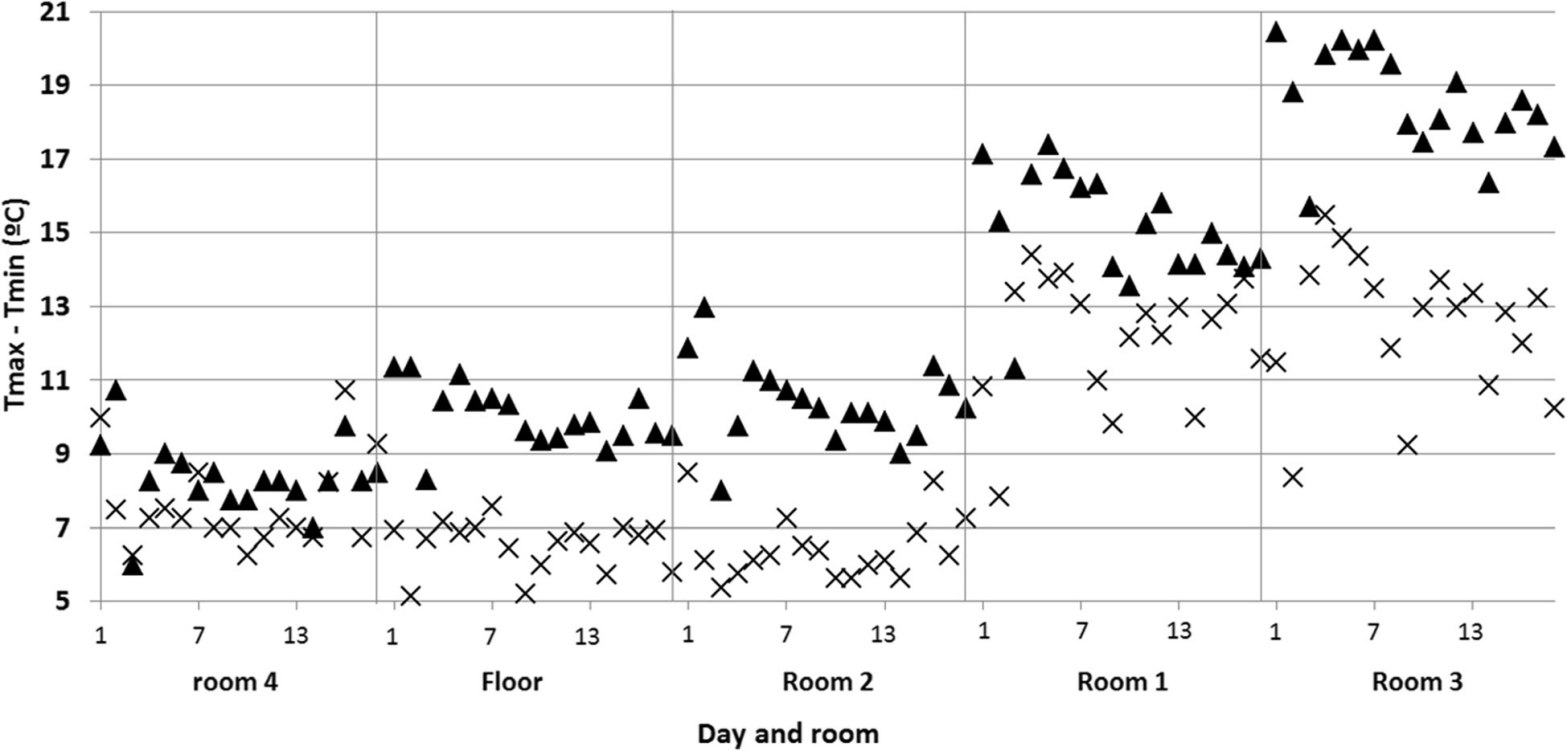
**Daily variations of temperature for each room.** Calculated as the difference between maximum and minimum temperatures recorded during the 18 days under study (filled triangles: 14–31 August 2008; crosses: 14–31 August 2010), averaged for probes in each room (except those in the floor).

The daily variation of temperature (DVT) for a given day was computed as the difference of the maximum (T_max_) and minimum (T_min_) recorded values. It is well known that DVT should be kept as low as possible for an optimum conservation of wall paintings. DVT was calculated for the 18 days under study (Figure 4). In 2010, the most stable conditions (i.e., lowest DVT) were found in rooms 2 and 4 as well as floor sensors (DVT ≈ 7°C). Room 4 also yielded a similar DVT in 2008 because the roof was maintained and the 18-day time frame was properly chosen to achieve an equal mean temperature in this room for both years.

In 2008, the lowest DVT was achieved in room 4, while data-loggers in room 2 and those on the floor yielded a DVT = 10.2°C. Thus, DVT underwent a reduction of about 4°C in room 2 caused by the roof change, becoming the lodging with better conditions for the frescoes. This room is delimited by four walls and presents a semi-confined environment more isolated from outside fluctuations, which would explain its low DVT in 2010. DVT was 15.1°C (room 1) and 18.5°C (room 3) in 2008, but it decreased to 12.3°C in 2010. Although this reduction is favorable from a preservation viewpoint, there is still a need to achieve a further decrease of DVT, trying to reach the microclimate of room 2. One option would be to insulate the roof with spray polyurethane foam. Another alternative, though more expensive, is to replace the undulating fiber cement sheets currently covering rooms 1–3 by foam-filled insulated roof sheets.

Although DVT is a good parameter to evaluate the effect of roof change, the opaque shelter has decreased the temperature in rooms 1 and 3 (Figure 3b) probably due to the different solar radiation incident on the walls through the roof. Preliminary measurements of direct light radiation were carried out in 2008 [23], and a detailed study about indirect light radiation in the rooms with the new coverage will be addressed as part of the on-going conservation project.

Results indicate that the roof change has improved the conditions for the preservation of wall paintings, but not enough. The goal would be to achieve a microclimate in rooms 1 and 3 similar to that in room 2. Thus, a general recommendation to reduce the deterioration of Pompeian frescoes in other houses would be to cover the lodgings with opaque roofs, preferentially containing thermal insulation. Another useful measure would be to avoid direct contact of sunshine radiation in summer by installing some kind of vertical curtains, shades or microperforated fabrics as a parapet hanging on the roof edge.

**Figure 5:**
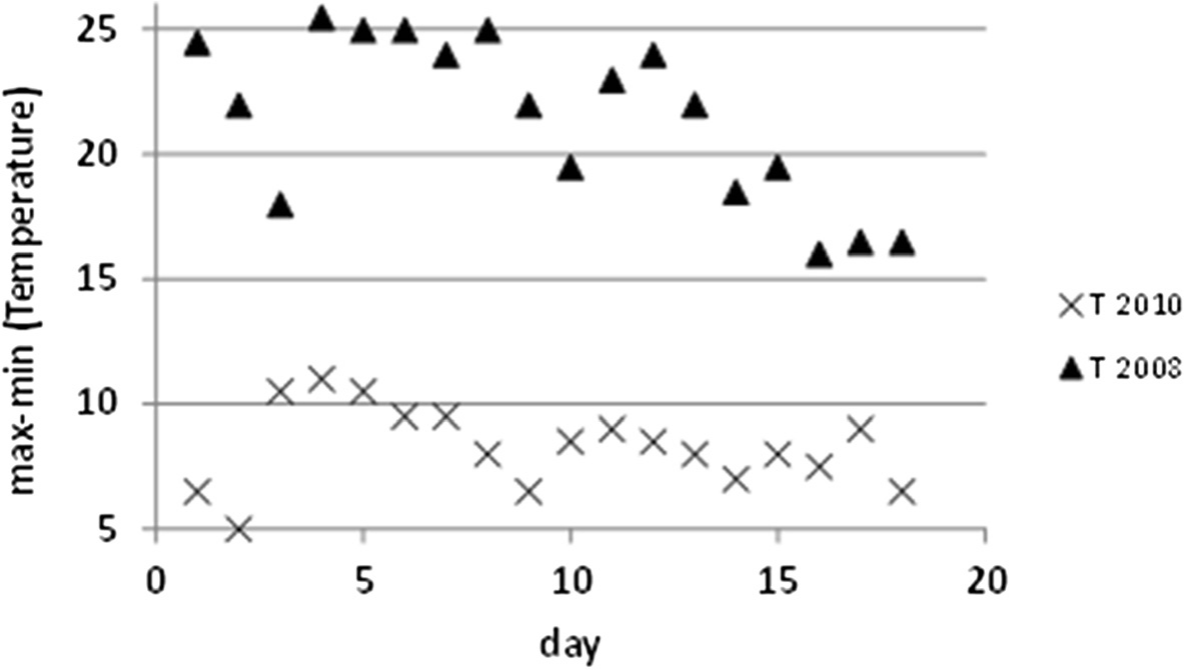
**Daily variations of temperature for data-logger #5 (mosaic).** Difference between maximum and minimum temperatures recorded during 18 days (filled triangles: 14–31 August 2008; crosses: 14–31 August 2010).

Probe #5 was installed inside the glass box protecting the mosaic in room 3. In 2008, the glass received direct sunshine through the transparent roof, causing a severe greenhouse effect with DVT up to 25°C (Figure 5), which is extremely harmful. This effect was eliminated with the roof change, resulting a DVT of about 7°C, which is similar as in the case of floor probes (Figure 4).

### Analysis of variance (ANOVA)

In order to further study the effect of roof change, different parameters were calculated for each RH data-logger and each day in both periods under study (14–31 August): maximum (RH_max_), minimum (RH_min_) and average. Daily averages of temperature, T_max_ and T_min_ values were computed as well. Different ANOVAs were carried out considering two factors: data-logger and year.

**Figure 6:**
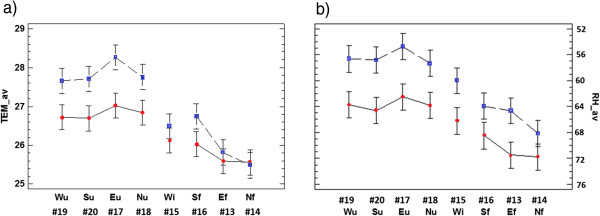
**ANOVA results (room 2): interaction plot.** Plot showing the effect of year (2008: blue squares; 2010: red circles) and data-logger on the daily mean temperatures (**a**) and RH (**b**) recorded in room 2 from 14^th^ to 31^st^ of August. Codes indicate the wall orientation (North, South, East or West) and the height (u: upper position; i: intermediate; f: floor level). For each data-logger, the average and 95% LSD interval is illustrated.

An effect of sensor height in room 2 is reflected by Figure 6a. Floor probes recorded lower temperatures with a similar mean in both years. By contrast, probes at the upper position (#17 - #20) registered higher values in 2008 due to the effect of solar radiation incident on the upper parts of walls before the roof change. The observed differences are statistically significant (α=0.05) because the LSD (Least Significant Difference) intervals do not overlap. The pattern of temperature according to sensor height is inversely related to the pattern of RH (Figure 6b) because higher temperatures imply lower RH and vice-versa. The observed differences of RH according to year are also statistically significant. Such clear effect of sensor height on thermohygrometric parameters is not so apparent in the other lodgings probably because room 2 is the only one delimited by four walls, which provides more stable conditions.

**Figure 7:**
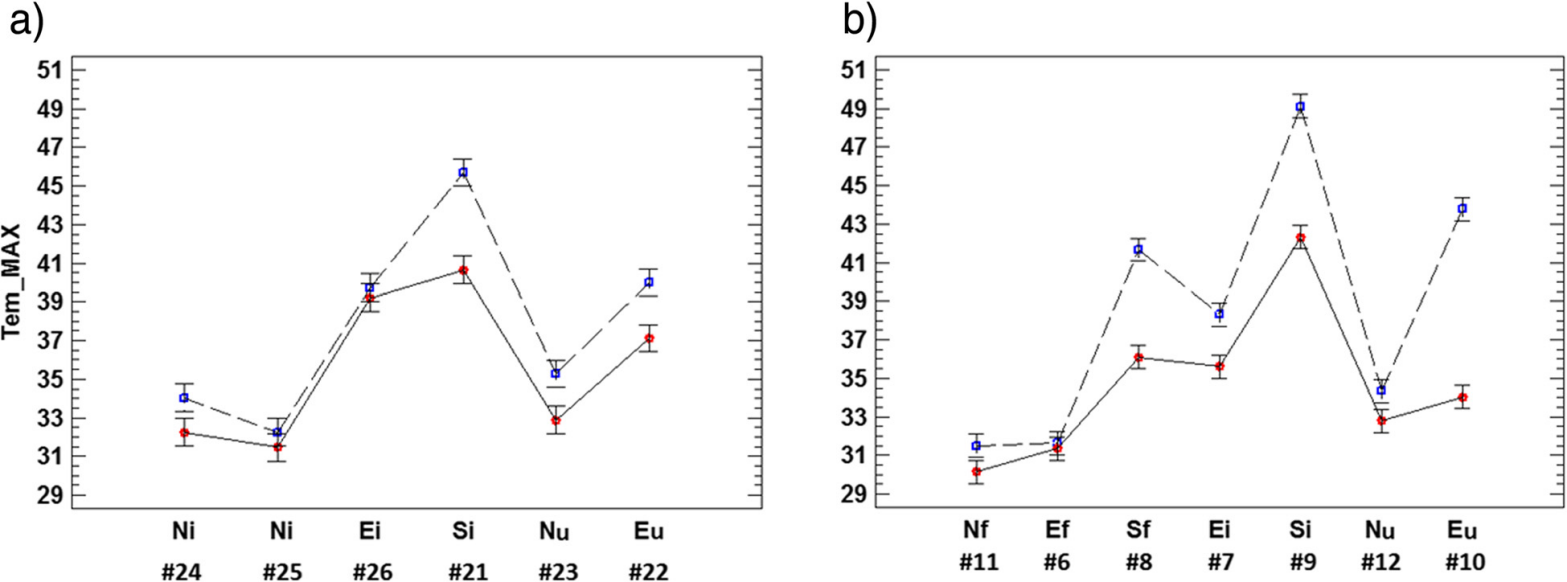
**ANOVA results of T**_**max **_**in rooms 1 and 3: interaction plot.** Plot (average and 95% LSD intervals) showing the effect of data-logger and year (2008: blue squares; 2010: red circles) on the daily maximum temperatures in room 1 (**a**) and room 3 (**b**) from August 14^th^ to 31^st^. Codes as in Figure 6.

The T_max_ (averaged for the 18-day period) of probes in rooms 1 and 3 is shown in Figures 7a and 7b, respectively. This parameter is remarkably different among probes in room 3, ranging from 31.5°C (#11) to 49.1°C (#9) in 2008 (Figure 7b). Such variability represents a serious risk for conservation purposes [27]. No significant differences between 2008 and 2010 are observed in probes that recorded the lowest T_max_ values (#6, #11, #12, #24, and #25). All of these data-loggers (except #6) are located on walls facing to the north, which is the orientation receiving less solar radiation. By contrast, the highest T_max_ values, particularly in 2008, were basically recorded by sensors facing to the south (#8, #9 and #21). The reduction of solar radiation incident on walls due to the opaque shelter has decreased T_max_ in rooms 1 and 3 (#8, #9, #10, and #21), particularly in those positions that received more sunshine.

**Figure 8:**
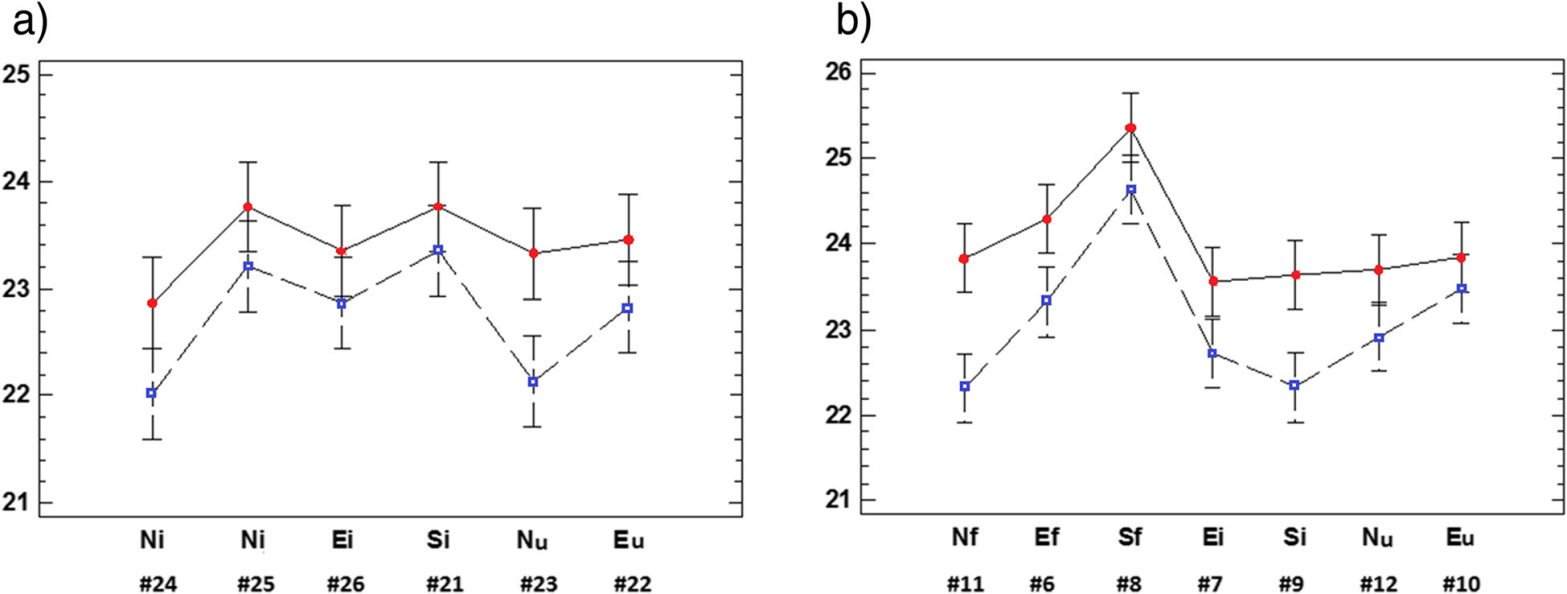
**ANOVA results of T**_**min **_**in rooms 1 and 3: interaction plot.** Plot of factors data-logger and year (2008: blue squares; 2010: red circles) on the daily minimum temperatures in room 1 (**a**) and room 3 (**b**). Codes as in Figure 6.

The change of shelter has also produced a slight increase of T_min_ in about 0.6°C in rooms 1 and 3 (Figure 8). The differences are not statistically significant for all probes because some LSD intervals overlap, but the parallel trend is apparent in both rooms. The decrease of T_max_ caused by the roof change and the slight increase of T_min_ results in a lower DVT in 2010 (Figure 4), which involves that the microclimate was more stable throughout the day and, hence, more appropriate for conservation purposes.

### Bivariate plots

**Figure 9:**
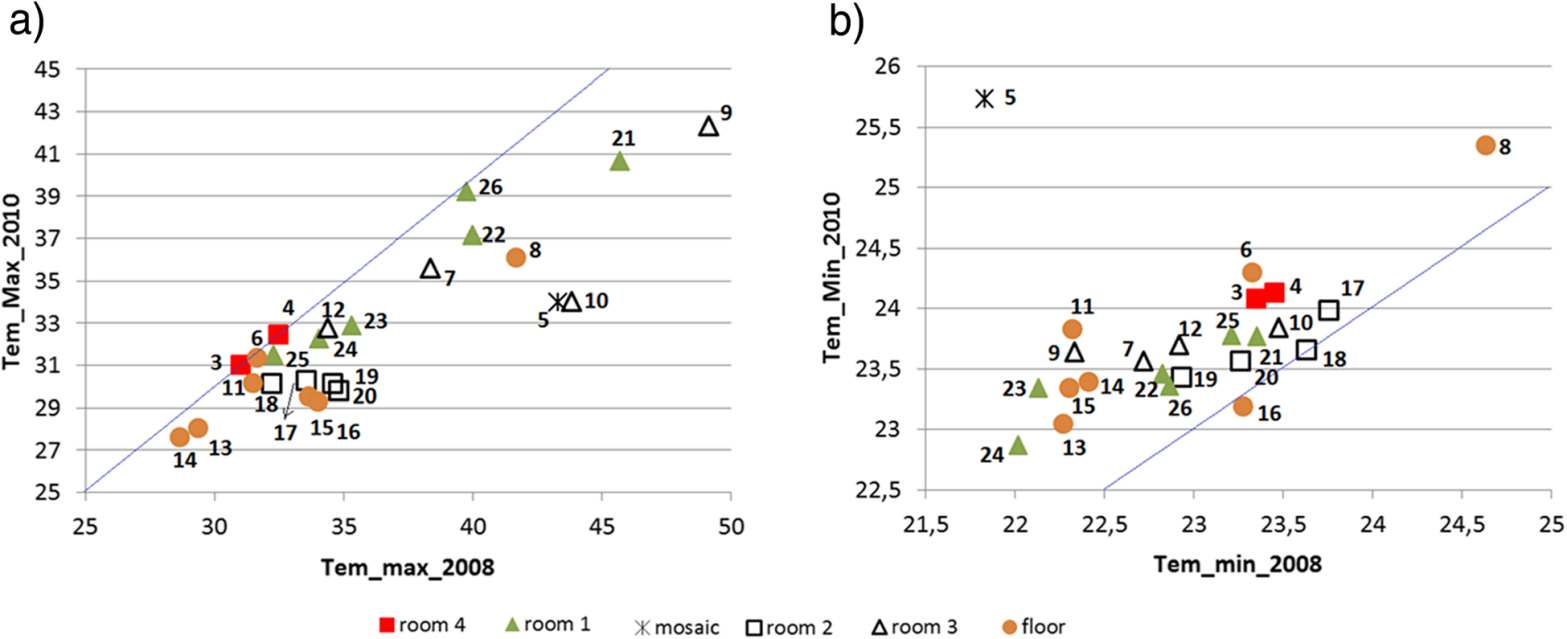
**Bivariate plot of daily maximum (T**_**max**_**) and minimum (T**_**min**_**) temperatures in 2010 *vs*. 2008.** Bivariate plot of T_max_ (**a**) and T_min_ (**b**) recorded from each probe in 2010 *vs*. 2008 (monitoring period: 14^th^ to 31^st^ of August). The tilted line is the bisector.

Figure 9a displays a scatterplot of T_max_ in 2010 *vs*. T_max_ in 2008. Probes #3 and #4 appear on the bisector line (i.e., equal mean values in both years), which indicates that the 18-day period was correctly selected. Some additional probes are also close to the bisector, but most of them yielded a lower T_max_ in 2010 as discussed above. The high T_max_ of #9, #10 or #21 is also reflected in Figure 7, but Figure 9a provides complementary information because all probes are depicted. The bivariate plot of T_min_ in 2010 *vs*. T_min_ in 2008 (Figure 9b) is consistent with Figure 8 and reveals that T_min_ was about 0.6°C higher in 2010 on average compared with 2008, except in the case of room 2 with 0.3°C of variation.

**Figure 10:**
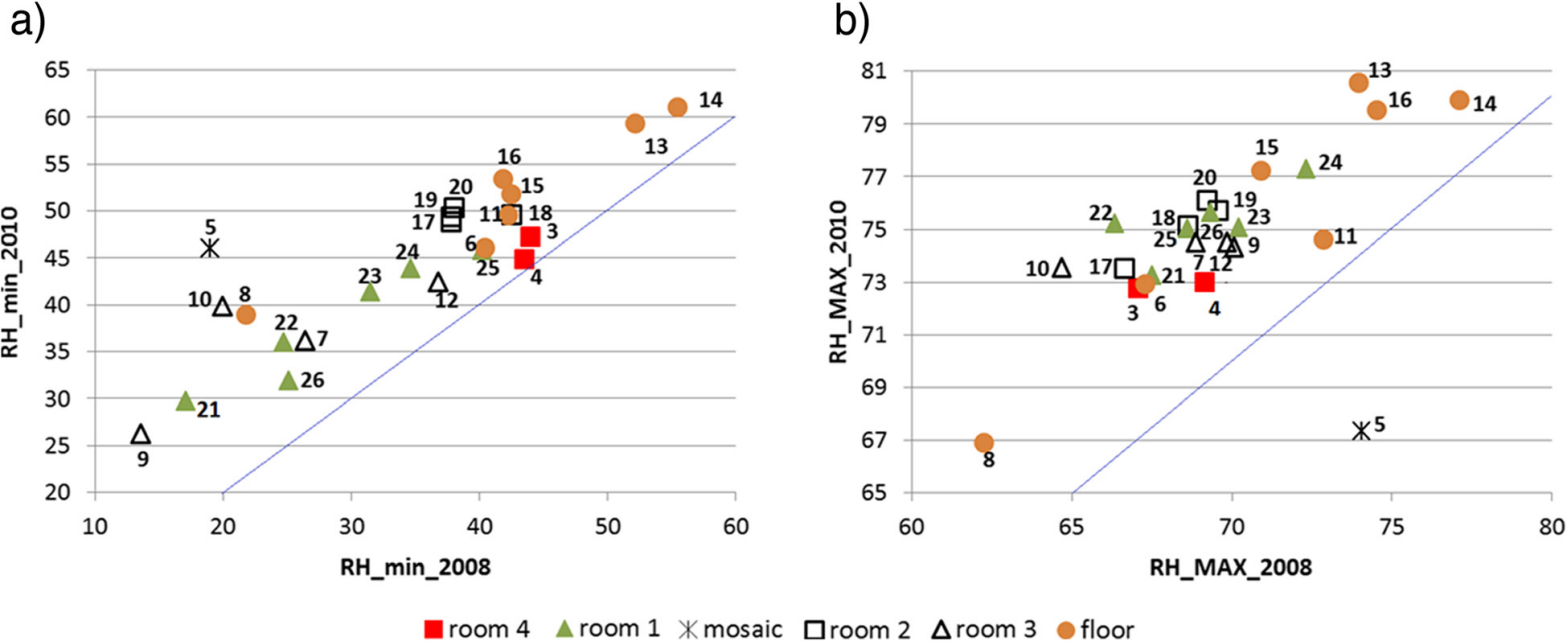
**Bivariate plot of daily minimum (RH**_**min**_**) and maximum (T**_**max**_**) relative humidities in 2010 *vs*. 2008.** Bivariate plot of RH_min_ (**a**) and RH_max_ (**b**) recorded from each probe in 2010 *vs*. 2008 (14^th^ to 31^st^ of August). The tilted line is the bisector.

RH and temperatures measured during one day by a given probe were negatively correlated according to Figure 3 because the maximum temperatures recorded at midday correspond to the minimum RH. Moreover, probes that yielded the highest temperatures (T_max_) also recorded the lowest RH (RH_min_), which was also reported in the previous study [24]. This inverse relationship between T_max_ and RH_min_ shows up by comparing Figures 9a and 10a because the relative position of probes in both figures is basically the same. There is also certain similarity between T_min_ (Figure 9b) and RH_max_ (Figure 10b).

In 2010, half of the probes registered RH_min_ values above 45%, but only two probes (#13 and #14) satisfied this condition in 2008 (Figure 10a). Taking into account that 45-60% is the recommended range of RH according to [27] for mural paintings, this result implies that the change of roof has improved the RH conditions from a conservation standpoint. In the case of RH_max_ (Figure 10b), the points follow a linear trend, and it can be deduced that RH increased around 6 units in 2010 with respect to 2008.

Probe #5 was affected by a severe greenhouse effect in 2008 that is no longer present in 2010, but it behaves as an outlier in Figures 9b and 10b, with an abnormal low RH_max_ and a high T_min_. The interpretation is uncertain because #5 was the only probe inside the glass box protecting the mosaic. The microclimate inside this box is totally confined and cannot be directly compared with ambient conditions in the rooms.

#### Efficiency of transparent *vs*. opaque shelters

A list of 222 covered archaeological sites in Italy is available in the literature (see annex of [26]), 38.7% of which are regarded as shelters with an intermediate efficiency and 59% as highly efficient. The tile roof of room 4 appears in this list as highly efficient, with a score of 7.1 on a 0–10 scale, while transparent shelters covering the other rooms scored 5.5 (intermediate). However, these scores were calculated using qualitative criteria not based on microclimate studies. In our opinion, such efficiency index may provide a rough guidance for archaeological sites curators, but it could be improved by taking into consideration parameters derived from multivariate microclimate monitoring studies. The present work may provide guidelines for a methodology aimed at comparing the efficiency of shelters in different locations.

### Experimental

#### Description and installation of data-loggers

The same set of RH and temperature data-loggers (models Hygrochron DS1923 [28] and Thermochron DS1922L [29], respectively) used in the previous study [24] was re-installed in Ariadne’s house in July 2010. According to the manufacturer (Maxim Integrated Products, Inc., Sunnyvale, CA), the accuracy is ±5% RH [28]. All data-loggers were calibrated prior to their installation in 2008 as described in [24]. Based on the calibration experiment, it was obtained that the temperature biases ranged from −0.44°C to +0.53°C (see Table 1 of [24]), which is nearly coincident with the accuracy range (±0.5°C) indicated by the manufacturer [28]. These biases were corrected as described in [24] to improve the accuracy of temperature measurements. Another calibration experiment was carried out in 2010 with RH sensors by comparing their measurements with reference values of a standard procedure based on aqueous solutions of two salts (NaCl and LiCl) [30], resulting a bias below 1% for all data-loggers. RH recordings were corrected taking into account the experimental biases to improve the accuracy.

Each probe was composed of one pair of DS1923 and DS1922L data-loggers assembled together by means of a PVC protective structure with a cylindrical shape (6 cm of diameter), which allows a convenient fixing to the wall. One measurement was recorded from each data-logger every 30 minutes, which involves 48 recordings per day. All probes were placed in the same positions as in the monitoring experiment of 2008 [24] in order to allow an appropriate data comparison of both years. The purpose was to locate data-loggers in the four rooms at three levels: floor (0 – 15 cm), intermediate (< 2 m) and upper position ( > 2 m) (Table 1).

### Statistical data analysis

Firstly, we computed the mean values recorded in 2010 by each data-logger at 0:00, 0:30, 1:00… and so on until 12:00 PM, resulting a daily mean trajectory. The calculation was performed with the set of 18 days under study (14–31 August), which were selected to achieve an equal mean temperature in room 4 in both years. All trajectories were visually inspected in order to detect abnormal peaks as those identified in the previous study [24]. Next, trajectories of probes in the same room were averaged, except those on the ground that were set aside (Figure 3). This procedure was also applied to data recorded in the same time frame of 2008, and the differences were discussed.

For each day and probe, the following parameters were computed: mean temperature, mean RH, T_max_, T_min_, RH_max_, and RH_min_. One important parameter frequently considered in the conservation of cultural heritage [27] is the daily variation of temperature (i.e., T_max_ − T_min_). This parameter was computed for the 18-day period of each year and it was averaged for probes in the same room, which provides useful information about the effect of roof change (Figure 4). In order to study if the differences among probes in the same room were statistically significant, different multifactor ANOVAs were performed with the key thermohygrometric parameters considering two factors: year (2008 or 2010) and probes (Figures 6, 7, 8). All ANOVA models were carried out with the software Statgraphics 5.1 [31].

Attempting to further characterize the differences among probes according to year, different bivariate plots (Figures 9 and 10) were obtained with T_max_, T_min_, RH_max_, and RH_min_ (averaged for the 18 days). They provide further information about the relationship between RH and temperature. RH values were discussed according to the guidelines for indoor mural paintings indicated by the Italian standard DM 10/2001 [27].

## Conclusions

The statistical analysis has revealed a considerable reduction of about 3.5°C in the maximum daily values reached in summer at rooms 1 and 3 caused by the roof change. Results show that this corrective measure adopted in 2009 has lowered the maximum temperatures and has also increased the RH and minimum temperatures, which entails an attenuation of daily variations of thermohygrometric conditions. Thus, the roof change has created a microclimate more stable and less harmful for the conservation of frescoes.

The steadiest ambient conditions were found in room 2, which is intuitively appealing because this room is delimited by four walls and the microclimate inside is more isolated from outdoor fluctuations. The roof change has enhanced the conditions in rooms 1 and 3 from a preservation standpoint, but they can be further improved to resemble those in room 2. For this purpose, several additional corrective measures are proposed.

An effect of sensor height was detected in room 2. The higher temperatures recorded there at the upper positions were due to diffuse solar radiation incident on walls through the transparent roof in 2008. In rooms 1 and 3, it was also found that sensors facing to the south recorded higher temperatures particularly in 2008, probably because the south orientation received more solar radiation before the roof change.

The methodology of data analysis applied here is also of interest for similar studies aimed at comparing thermohygrometric data recorded in different periods, because it is necessary to avoid the confusion of effects that appears when the average conditions of the periods are different.

## Competing interests

The authors declare that they have no competing interests.

## Authors’ contributions

All authors contributed equally to this work. PM is a researcher developing a PhD thesis about statistical monitoring for preventive conservation of cultural heritage; FJGD was responsible for the design of probes and their implementation, and MZ provided statistical assistance with the data analysis. All authors read and approved the final manuscript.
